# The effect of the speed and range of motion of movement on the hyperemic response to passive leg movement

**DOI:** 10.14814/phy2.14064

**Published:** 2019-04-19

**Authors:** Jayson R. Gifford, Travis Bloomfield, Trevor Davis, Amy Addington, Erin McMullin, Taysom Wallace, Meagan Proffit, Brady Hanson

**Affiliations:** ^1^ Department of Exercise Sciences Brigham Young University Provo Utah; ^2^ Program of Gerontology Brigham Young University Provo Utah

**Keywords:** Endothelial function, exercise blood flow, movement speed, passive leg movement, range of motion

## Abstract

Passive leg movement (PLM)‐induced hyperemia is used to assess the function of the vascular endothelium. This study sought to determine the impact of movement speed and range of motion (ROM) on the hyperemic response to PLM and determine if the currently recommended protocol of moving the leg through a 90° ROM at 180°/sec provides a peak hyperemic response to PLM. 11 healthy adults underwent multiple bouts of PLM, in which either movement speed (60–240°/sec) or ROM (30–120° knee flexion) were varied. Femoral artery blood flow (Doppler Ultrasound) and mean arterial pressure (MAP; photoplethysmography) were measured throughout. Movement speed generally exhibited positive linear relationships with the hyperemic response to PLM, eliciting ~15–20% increase in hyperemia and conductance for each 30°/sec increase in speed (*P* < 0.05). However, increasing the movement speed above 180°/sec was physically difficult and seemingly impractical to implement. ROM exhibited curvilinear relationships (*P*<0.05) with hyperemia and conductance, which peaked at 90°, such that a 30° increase or decrease in ROM from 90° resulted in a 10–40% attenuation (*P* < 0.05) in the hyperemic response. Alterations in the balance of antegrade and retrograde flow appear to play a role in this attenuation. Movement speed and ROM have a profound impact on PLM‐induced hyperemia. When using PLM to assess vascular endothelial function, it is recommended to perform the test at the traditional 180°/sec with 90° ROM, which offers a near peak hyperemic response, while maintaining test feasibility.

## Introduction

Originally developed to partition the roles of mechanical movement and muscle metabolism in exercise hyperemia (Wray et al. 2005), the passive leg movement (PLM) technique has since proven to be an insightful indicator of peripheral vascular endothelial function (Gifford and Richardson 2017). Indeed, the magnitude of hyperemia elicited by passively moving an individual's leg has been used to characterize the vascular health of various populations ranging from healthy young adults (Groot et al. 2016) to the elderly (Groot et al. 2015), and various other populations (Witman et al. 2015). While published recommendations for PLM procedures currently suggest moving the leg through a 90° ROM at 180°/sec (i.e.*,* 60 cycles per minute; CPM) (Gifford and Richardson 2017), there is no published evidence justifying this specific combination of speed and ROM. Consequently, it is unclear if this traditional protocol is optimal for observing the function of the peripheral vasculature and what effect, if any, variation in the movement speed or ROM of the PLM may have on the hyperemia observed.

To date, no studies have examined the impact of movement speed on PLM‐induced hyperemia, but several studies performed with active exercise suggest that leg blood flow may be sensitive to movement speed (Ferguson et al. 2001; Osada and Rådegran 2002; Sjøgaard et al. 2002). For example, when performing knee extension exercise at a given power output, a faster cadence (e.g.*,* contractions or cycles per minute, CPM) generally elicits greater leg blood flow than a slower cadence (Ferguson et al. 2001; Sjøgaard et al. 2002). Nevertheless, it is not clear if such is the case during PLM, which does not involve the large increase in metabolism (Hellsten et al. 2008) typical of active exercise (Gifford et al. 2016). While the effect of ROM on PLM‐induced hyperemia has yet to be described, there is evidence demonstrating that the peripheral vascular system is sensitive to changes in knee joint angle or movement ROM. For example, in 2012, McDaniel et al. (2012) observed that resting leg blood flow is lower when the knee is in a flexed position compared to an extended position, in part due to augmented retrograde flow in the flexed position. If such is the case during PLM of varying ROM's remains to be seen.

Recently, Kruse et al. (2018) investigated the role of movement speed and ROM in PLM‐induced changes in central factors, like heart rate and mean arterial pressure (MAP). Performing PLM at various speeds and ROM, they found that the changes in heart rate and MAP were related to the speed and ROM of the PLM, with greater values eliciting greater changes in central factors. As the changes in central factors, like heart rate and MAP, generally occur alongside (Broxterman et al. 2017), if not in response to (Gifford and Richardson 2017), the changes in leg blood flow and vascular conductance during PLM, it seems very likely that movement speed and ROM impact the magnitude of PLM‐induced hyperemia.

With the magnitude of hyperemia elicited by PLM being used to assess vascular health, it is important to know if variations in how the test is administered can impact the magnitude of hyperemia and, consequently, the interpretation of a person's vascular health. Evidence suggests that movement speed and ROM may have an impact on PLM‐induced hyperemia, but the extent to which this is the case has not been determined. Furthermore, it is also currently unclear if the traditional protocol (Gifford and Richardson 2017) of moving the leg through a 90° ROM at a speed of 180°/sec is optimal for observing the peak function of the peripheral vasculature. Therefore, the purpose of this study was to document the separate effects of movement speed and ROM on PLM‐induced hyperemia and to determine if the currently recommend protocol of moving the leg through a 90° ROM at a speed of 180°/sec provides a peak, or near peak, hyperemic response to PLM.

## Methods

### Subjects

Following approval by the Institutional Review Board at Brigham Young University and, and in accordance with the *Declaration of Helsinki*, 10 young (22.5 ± 0.7), healthy adults (3 female, 7 male) with a lean body mass index (BMI: 23 ± 0.6 kg/m^2^) were recruited for this study. Participants reported being moderately‐to‐highly active according to the International Physical Activity Questionnaire (Helmerhorst et al. 2012) and being free from medications with known cardiovascular effects.

### Experimental protocol

Participants reported to the laboratory well‐rested, having abstained from vigorous exercise (≥24 h), alcohol (≥24 h) and caffeine (≥12 h) and having fasted for at least 4 h on two separate occasions. Females were studied during the first 7 days of their menstrual cycle (Gifford and Richardson 2017).

Each visit involved multiple trials of PLM with methods that followed the previously published recommendations for PLM (Gifford and Richardson 2017), with the exception of how fast (i.e., angular velocity) or how far (i.e., ROM) the leg was moved during PLM. Specifically, upon arrival to the laboratory the participants’ right leg was placed in a knee brace that could be adjusted to allow different ROM of the knee joint. Participants then underwent multiple familiarization trials to ensure maximum relaxation during the movement. Participants were seated upright, with their legs extended in front of them for at least 20 min before measurements occurred. Leg blood flow (LBF) measurements were assessed 2–5 cm proximal to the bifurcation common femoral artery with Doppler ultrasound (Logiq E, General Electric Medical Systems, Milwaukee, WI) during 1 min of baseline, and during passive knee flexion‐extension with a trained‐member of the research team manually moving the participants’ leg through the desired ROM at the desired angular velocity. Measurements of arterial blood velocity and vessel diameter were assessed with the ultrasound system operating in duplex mode with a B‐mode imaging frequency of 12 MHz and a Doppler frequency of 4 MHz. All blood velocity measurements were assessed at an insonation angle of ≤60°. Artery diameter was taken as the average of 5 measurements made during baseline at a perpendicular angle along the central axis of the insonated area during end diastole using software on the ultrasound system. Beat‐by‐beat mean arterial pressure (MAP) was measured on a subset of participants throughout with finger photoplethysmography (CNAP, CNS systems, Austria).

### Testing the effect of movement speed on PLM‐induced hyperemia

During one visit the effect of movement speed (i.e., angular velocity) on PLM‐induced hyperemia was investigated by varying the angular velocity of the leg as it moved through a 90° ROM. Note that the methods described above were followed in this session, apart from varying the angular velocity (i.e., cycles per minute, CPM) of the movement. The movement speeds investigated were 60°/sec (i.e. 20 CPM), 120°/sec (i.e., 40 CPM), 180°/sec (i.e., 60 CPM), and 240°/sec (i.e., 80 CPM) in randomized order with 10 min rest in between each trial. Each PLM trial was performed long enough to have at least 60 sec of PLM, and 60 total movements. For example, while the 180°/sec PLM, which completes 60 movement cycles in 60 sec, was only performed for 60 sec, the 60°/sec PLM was continued beyond for 180 sec, in order to determine the effect of the exposure to 60 movement cycles of PLM, as well as the effect of 60 sec of exposure to PLM, on leg blood flow.

### Testing the effect of ROM on PLM‐induced hyperemia

On a separate visit, multiple trials of PLM were performed which varied in the ROM of the movement at a fixed angular velocity of 60°/sec using the methods described above (Gifford and Richardson 2017). Specifically, PLM‐induced hyperemia was assessed moving the leg through 30°, 60°, 90°, or 120° of knee flexion/extension in a randomized order with ~10 min of rest between each trial. Each PLM trial was performed long enough to expose the leg to at least 60 sec of PLM, as well as 60 total movements. Note that the movement speed in these ROM trials was fixed at a rate slower than the traditional 180°/sec (Gifford and Richardson 2017) to facilitate the movement of the leg through the greater ROM required during the 120° trial.

### Data analysis

Time averaged mean blood velocity (TAmean) was analyzed as total blood velocity, and as its components: antegrade and retrograde blood velocity. Leg blood flow was calculated as:

$$\text{Blood Flow}=\pi {\left(\frac{\text{artery diameter}}{2}\right)}^{2}*\text{TAmean}.$$

Vascular conductance was calculated as the quotient of femoral artery blood flow divided by simultaneous MAP. Blood flow and vascular conductance during the first 60 sec of PLM was analyzed on a second‐by‐second basis. A 3 sec rolling average was subsequently applied to smooth these data. Blood flow and vascular conductance during baseline and after the first 60 sec of PLM were analyzed in 12 sec averages, as pilot data indicated that flow was fairly stable during these times. As previously described (Gifford and Richardson 2017), the magnitude of hyperemia was quantified in terms of the peak change (ΔPeak) in blood flow or conductance elicited by each trial and by the total hyperemia, in terms of leg blood flow and vascular conductance, elicited by each trial (i.e., area under the curve, AUC) over the course of 60 sec (AUC_60sec_). As ΔPeak and AUC_60sec_ have been reported to be largely attenuated by nitric oxide synthase inhibition, these metrics are commonly reported as indices of endothelial function and NO bioavailability (Mortensen et al. 2012; Trinity et al. 2012). Since the different movement speeds and ROM completed movement cycles at different rates (e.g., 20–80 CPM), the total hyperemic response for 60 completed movement cycles of PLM (i.e., AUC_60cycles_) was also determined by continuing PLM until each condition completed a total of 60 movement cycles to determine if the effect of ROM or movement speed on the hyperemic response was merely a product of completing more or less movement cycles during the traditional 60 sec measurements.

### Statistical analyses

Repeated measures ANOVA were used to determine the effect of movement speed and ROM on PLM‐induced hyperemia. In the event of a significant omnibus, Tukey's least significant difference post hoc test was used to determine which speeds or ROM significantly differed from each other (SPSS version 24. Chicago, IL). Mixed model analysis (Harrison et al. 2018) (JMP Pro, version 14. Carey, NC) was utilized to determine and describe the relationship between movement speed, ROM and the hemodynamic variables of interest. In this modeling technique each subject's data were entered as a random effect, with random slopes and intercepts, to account for correlation/dependence between repeated measures. Subsequently, the curve of best fit was determined for each subject, which was then used to model the curve of best fit for the entire group of subjects. Alpha was set at 0.05 a priori. Data are represented as mean ± SE.

## Results

### Effect of movement speed on PLM‐induced hyperemia

**Figure 1:**
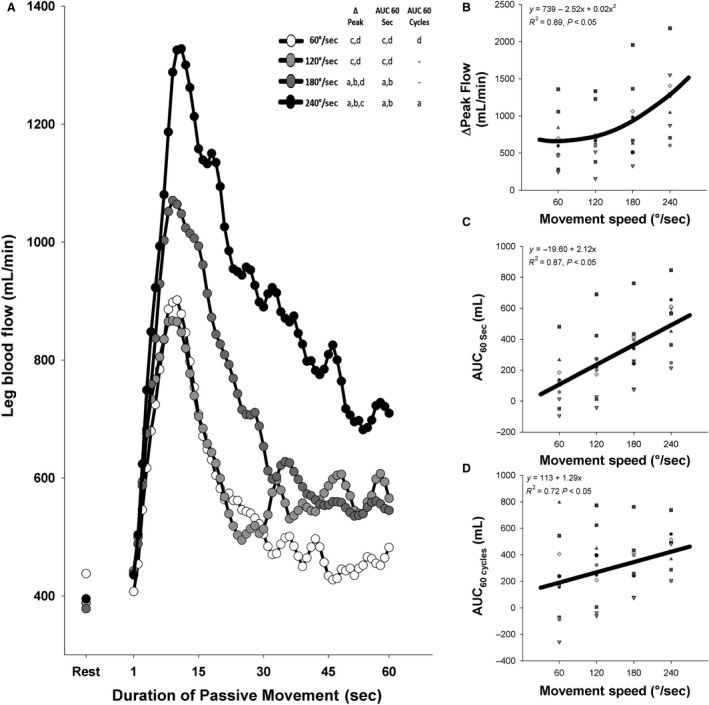
Effect of the speed of passive leg movement (PLM) on the hyperemic response to PLM. (A) Average leg blood flow response to PLM at different movement speeds. Error bars were not included for clarity. (B) Relationship between movement speed and peak change (ΔPeak) responses in blood flow elicited by PLM. (C) Relationship between movement speed and area under the curve during 60 sec (AUC_60sec_) of PLM representing the total hyperemic response during 60 sec of PLM. (D) Relationship between movement speed and area under the curve during 60 cycles (AUC_60cycles_) of PLM, representing the total hyperemic response during 60 cycles of PLM. “a”: significantly different than 60°/sec. “b”: significantly different than 120°/sec. “c”: significantly different than 180°/sec. “d”: significantly different than 240°/sec. “‐”: not significantly different. CPM: cycles per minute. In panels B–D the thick black line represents the curve of best fit between the individual hyperemic responses and movement speed.

As illustrated in Figure 1A, passively moving the subjects’ leg through a 90° range of motion at different movement speeds had a marked effect on the hyperemic response to PLM, with the ΔPeak, AUC_60sec_, and AUC_60cycles_ of several speeds significantly differing from those of other speeds (See Fig. 1A for which conditions significantly differed from one another). As illustrated in Figure 1B, mixed model analysis revealed that the peak change in blood flow during PLM was strongly related to movement speed in a positive curvilinear fashion (*y* = 739 − 2.52*x* + 0.02*x* ^2^, *R* ^2^ = 0.89, *P* < 0.05). The AUC_60sec_ (*y* = −19.60 + 2.12*x*,* R* ^2^ = 0.87, *P* < 0.05) and AUC_60cycles_ (*y* = 113 + 1.29*x*,* R* ^2^ = 0.72, *P* < 0.05) were also significantly related to movement speed, but in a positive, linear manner.

**Figure 2:**
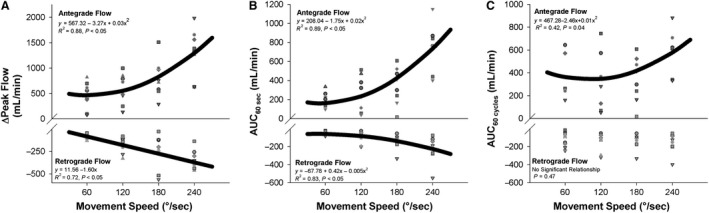
Effect of the speed of passive leg movement (PLM) on antegrade and retrograde blood flow during PLM. (A) Relationship between movement speed and peak change (ΔPeak) in antegrade and retrograde blood flow elicited by PLM. (B) Relationship between movement speed and area under the curve during 60 sec (AUC_60 sec_) of PLM for antegrade and retrograde flow. (C) Relationship between movement speed and area under the curve during 60 cycles (AUC_60 cycles_) of PLM. Note that the thick black line represents the curve of best fit between the individual hyperemic responses and movement speed.

With movement speed exhibiting a significant effect on the various indices of total blood flow, its relationship with antegrade and retrograde flow was subsequently examined. As illustrated in Figure 2A, movement speed significantly related to the ΔPeak in antegrade flow in a positive curvilinear manner (*y* = 567.32 − 3.27*x* + 0.03*x* ^2^, *R* ^2^ = 0.88, *P* < 0.05), while the ΔPeak of retrograde flow was related to movement speed in a linear manner *y* = 11.56 − 1.60*x*,* R* ^2^ = 0.72, *P* < 0.05) with greater movement speeds eliciting greater retrograde (i.e., negative) flow. The AUC_60sec_ for both antegrade (*y* = 208.04 − 1.75*x* + 0.02*x* ^2^, *R* ^2^ = 0.89, *P* < 0.05) and retrograde flow (*y* = −67.78 + 0.42*x* − 0.005*x* ^2^, *R* ^2^ = 0.83, *P* < 0.05) were related to movement speed in a curvilinear fashion, with greater speeds eliciting greater antegrade and retrograde flow. The AUC_60cycles_ for antegrade flow (*y* = 467.28 − 2.46*x* + 0.01*x* ^2^, *R* ^2^ = 0.42, *P* < 0.05) was also related to movement speed, while the AUC_60cycles_ for retrograde was not significantly related to movement speed (*P* = 0.47).

**Figure 3:**
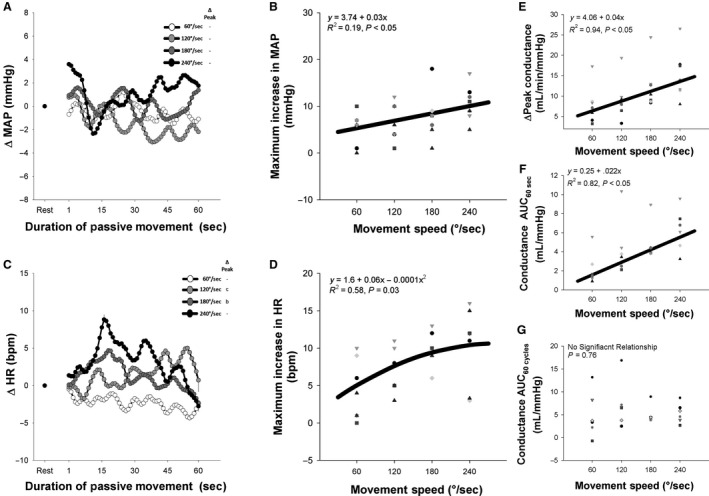
Effect of the speed of passive leg movement (PLM) on mean arterial pressure (MAP) heart rate (HR) and vascular conductance during PLM. (A) Average MAP response to PLM at different movement speeds. (B) Relationship between movement speed and peak increase in MAP during PLM. (C) Average HR response to PLM at different movement speeds. (D) Relationship between peak increase in HR during PLM and movement speed. (E) Relationship between movement speed and peak change (ΔPeak) in conductance during PLM. (F) Relationship between movement speed and area under the curve during 60 sec (AUC_60sec_) of PLM. (G) Relationship between movement speed and area under the curve during 60 cycles (AUC_60cycles_) of PLM. “a”: significantly different than 60°/sec. “b”: significantly different than 120°/sec. “c”: significantly different than 180°/sec. “d”: significantly different than 240°/sec. “‐”: not significantly different. Note that the thick black line represents the curve of best fit between the individual hyperemic responses and movement speed.

Beat‐by‐beat blood pressure and heart rate (HR) were measured in a subset of participants (*n* = 7) to explore the role of central factors in the relationship between movement speed and the hyperemic response to PLM. As illustrated in Figure 3A and B, the maximum increase in MAP was related to movement speed (Fig. 3B, *R* ^2^ = 0.19, *P* < 0.05), with greater speeds eliciting a greater increase in pressure during the movement. Notably, the transient decrease in MAP typically observed during PLM (Trinity et al. 2015) was consistent between speeds (*P* > 0.05), averaging approximately −6 ± 1 mmHg for each speed. The change in HR above resting values was also positively related to movement speed (Fig. 3C and D, *R* ^2^ = 0.58, *P* < 0.05). To determine if the relationship between movement speed and PLM‐induced hyperemia was dependent on central effects, the hyperemic response was subsequently considered in terms of peripheral vascular conductance. As illustrated in Figure 3E and F, the relationships between movement speed and ΔPeak (*R* ^2^ = 0.94, *P* < 0.05) and AUC_60sec_ (*R* ^2^ = 0.82, *P* < 0.05) persist when considered in terms of vascular conductance, both exhibiting strong positive linear relationships with movement speed.

### Effect of movement range of motion on PLM‐induced hyperemia

**Figure 4:**
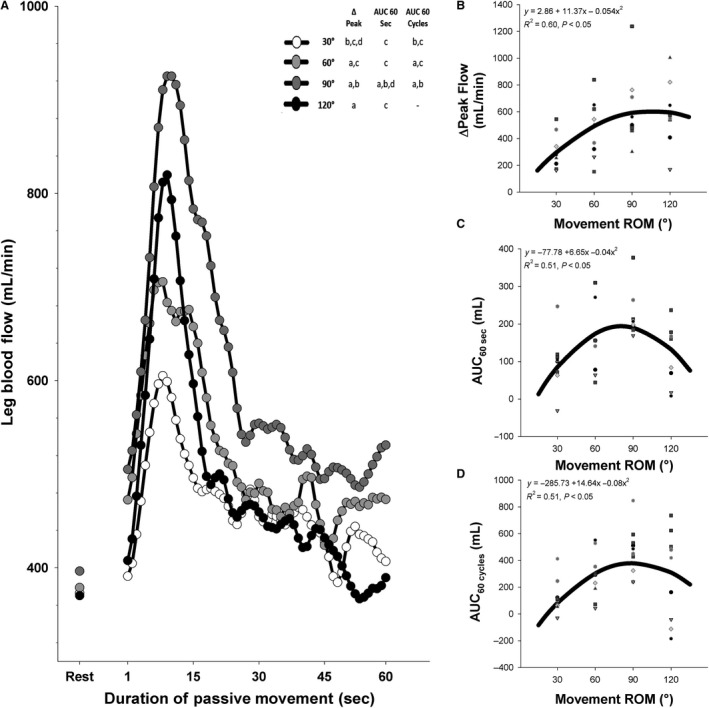
Effect of the range of motion (ROM) of passive leg movement (PLM) on the hyperemic response to PLM. (A) Average leg blood flow response to PLM at different ROM's. Error bars were not included for clarity. (B) Relationship between movement ROM and peak change (ΔPeak) in blood flow elicited by PLM. (C) Relationship between movement ROM and area under the curve during 60 sec (AUC_60sec_) of PLM. (D) Relationship between movement ROM and area under the curve during 60 cycles (AUC_60cycles_) of PLM. “a”: significantly different than 30°. “b”: significantly different than 60°. “c”: significantly different than 90°. “d”: significantly different than 120°. “–”: not significantly different from any condition. Note that the thick black line in panels B–D represents the curve of best fit between the individual hyperemic responses and movement speed.

As illustrated in Figure 4A, passively moving the subjects’ leg through different ranges of motion at a fixed movement speed (60°/sec) had a marked effect on the hyperemic response to PLM, with the ΔPeak, AUC_60sec_, and AUC_60cycles_ of several ROM significantly differing from those of other ROM (See Fig. 4A for which conditions significantly differed from one another). As illustrated in Figure 4B, a mixed model analysis revealed that the peak change in blood flow during PLM was strongly related to movement ROM in a positive curvilinear fashion (*y* = 2.86 + 11.37*x*−0.054*x* ^2^, *R* ^2^ = 0.60, *P* < 0.05). The AUC_60sec_ (*y* = −77.78 + 6.65*x* − 0.04*x* ^2^, *R* ^2^ = 0.51, *P* < 0.05) and AUC_60cycles_ (*y* = −285.73 + 14.64*x* − 0.08*x* ^2^, *R* ^2^ = 0.51, *P* < 0.05) were also significantly related to movement ROM in a curvilinear manner. Notably, as illustrated in Figure 4B–D, the relationship between ROM and the hyperemic response to PLM generally peaked or plateaued around 90° ROM.

**Figure 5:**
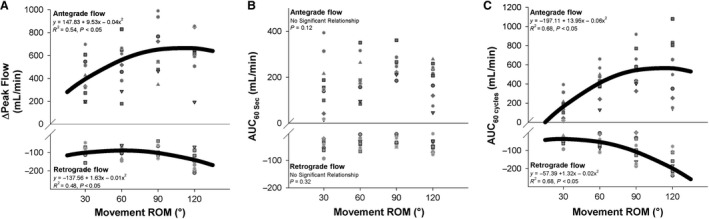
Effect of the range of motion (ROM) of passive leg movement (PLM) on antegrade and retrograde blood flow during PLM. (A) Relationship between movement ROM and peak change (ΔPeak) in antegrade and retrograde blood flow elicited by PLM. (B) Relationship between movement ROM and area under the curve during 60 sec (AUC_60sec_) of PLM for antegrade and retrograde flow. (C) Relationship between movement ROM and area under the curve during 60 cycles (AUC_60cycles_) of PLM. Note that the thick black line represents the curve of best fit between the individual hyperemic responses and movement speed.

With movement ROM exhibiting a significant effect on the various indices PLM‐induced hyperemia, its relationship with antegrade and retrograde flow was subsequently examined. As illustrated in Figure 5A, movement ROM significantly related to the ΔPeak in antegrade flow (*y* = 147.83 + 9.53*x* − 0.04*x* ^2^, *R* ^2^ = 0.54, *P* < 0.05) and retrograde flow (*y* = −137.56 + 1.63*x* − 0.01*x* ^2^, *R* ^2^ = 0.48, *P* < 0.05) in a curvilinear manner. Notably, the AUC_60sec_ for both ante grade and retrograde flow were unrelated to movement ROM (Fig. 5B, *P* > 0.05). Meanwhile the AUC_60cycles_ for antegrade flow (*y* = −197.11 + 13.95*x*−0.06*x* ^2^, *R* ^2^ = 0.68, *P* < 0.05) and retrograde flow (*y* = −57.39 + 1.32*x* − 0.02*x* ^2^, *R* ^2^ = 0.68, *P* < 0.05) were both related to movement ROM in a curvilinear fashion (Fig. 5C).

**Figure 6:**
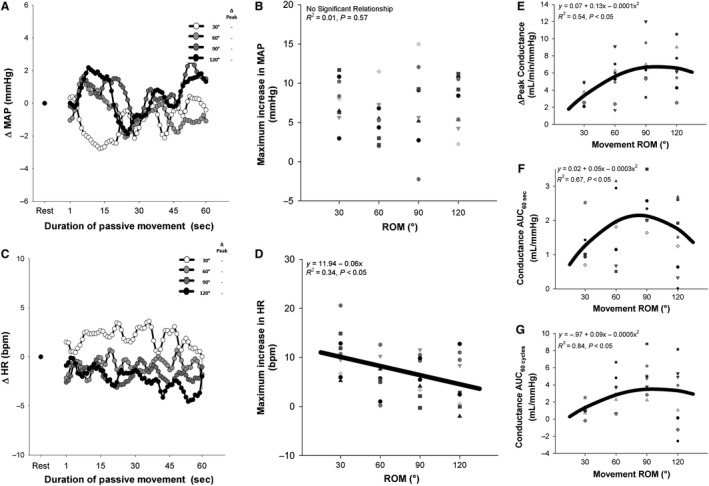
Effect of the range of motion (ROM) of passive leg movement (PLM) on mean arterial pressure (MAP) heart rate (HR) and vascular conductance during PLM. (A) Average MAP response to PLM at different movement ROM. (B) Relationship between movement ROM and peak increase in MAP during PLM. (C) Average HR response to PLM at different movement ROM. (D) Relationship between movement ROM and the peak increase in HR during PLM. (E) Relationship between movement ROM and peak change (ΔPeak) in conductance during PLM. (F) Relationship between movement ROM and area under the curve during 60 sec (AUC_60sec_) of PLM. (G) Relationship between movement ROM and area under the curve during 60 cycles (AUC_60cycles_) of PLM. “a”: significantly different than 30°. “b”: significantly different than 60°. “c”: significantly different than 90°. “d”: significantly different than 120°. “–”: not significantly different from any condition. Note that the thick black line represents the curve of best fit between the individual hyperemic responses and movement ROM.

As illustrated in Figure 6A and B, the peak increase in MAP, measured in a subset of participants (*n* = 9) was unrelated to movement ROM (*P* = 0.57). Meanwhile, the change in HR above resting values was negatively related to movement ROM (Fig. 5C and D, *R* ^2^ = 0.34, *P* < 0.05), with the smallest ROM eliciting the greatest increase in HR. As illustrated in Figure 5E–G, the relationships between movement ROM and ΔPeak (*R* ^2^ = 0.54, *P* < 0.05), AUC_60sec_ (*R* ^2^ = 0.67, *P* < 0.05) and AUC_60cycles_ (*R* ^2^ = 0.84, *P*<0.05) persist when considered in terms of vascular conductance, exhibiting curvilinear relationships that either peak or plateau around 90° ROM. Note that no differences in arterial diameter (*P* > 0.05) or resting blood flow (*P* > 0.05) were observed for the repeated measures in either the ROM visit or movement speed visit.

## Discussion

Passive leg movement‐induced hyperemia is emerging as a useful test to discern differences in peripheral vascular function between different populations and under different conditions. The purpose of this study was to document the separate effects of movement speed and ROM on PLM‐induced hyperemia and to determine if the currently recommend protocol of moving the leg through a 90° ROM at a speed of 180°/sec provides a peak, or near peak, hyperemic response to PLM. As will be discussed below, this study indicates that PLM‐induced hyperemia is very sensitive to both movement speed and movement ROM, and that the current protocol of 90° ROM and 180°/sec elicits near‐peak hyperemic responses to discern the function of the peripheral vasculature.

### Effect of movement speed on PLM‐induced hyperemia

To date, most studies utilizing PLM‐induced hyperemia to assess vascular function have been performed at a rate of 180°/sec (i.e. 60 CPM through a 90° ROM) (Gifford and Richardson 2017). While there is conformity in using a rate of 180°/sec, there currently is little justification for using this as the standard rate. As illustrated in Figure 1, movement speed has a clear effect on the hyperemia elicited by PLM. In general, increased movement speed is related to an increased hyperemic response to PLM. As illustrated in Figure 2, movement speed is related to both antegrade and retrograde flow, with greater speeds eliciting greater antegrade and retrograde flow.

While Figures 1 and 2 makes it clear that the hyperemic response to PLM is sensitive to differences in movement speed, based upon those data it is unclear if this is due to the response of the vasculature or the response of central factors that culminate in an increased perfusion pressure (i.e., MAP) to the vasculature. Therefore, the response of the vasculature within the leg was further isolated by simultaneously measuring blood flow, MAP, and heart rate during PLM in a subset of subjects (*n* = 7). As illustrated in Figure 3A and B, movement speed was associated with an augmented increase in MAP (Fig. 3B) and heart rate (Fig. 3D), confirming findings from Kruse et al. that increased movement speed during PLM is associated with an exaggerated central response.

With increased movement speed being associated with an augmented central response, it is possible that the increased hyperemic response observed during the faster movement speeds is merely due to an increased perfusion pressure rather than any change in the response of the vasculature in the leg. Nevertheless, as illustrated in Figure 3E–F, despite exhibiting visually greater variability than the when expressed as flow (likely due to variability in the MAP measurement), the relationship between movement speed and the hyperemic response to PLM persists when flow is normalized for MAP (i.e. vascular conductance). Thus, the effect of movement speed on PLM‐induced hyperemia is not merely the result of an increased central response, but is also likely the consequence of speed‐induced changes in the response of the local vasculature itself.

The effect of movement speed on the hyperemic response to PLM is quite meaningful, with a 30°/sec deviation (i.e. 10 cycles per minute change) from the standard 180°/sec protocol speed eliciting a 15–21% change in ΔPeak flow and AUC_60sec_ (Fig. 1B and C), which approaches the % difference observed between populations like active and sedentary individuals (Gifford and Richardson 2017). Thus, if movement speed was not carefully controlled when administering a test, a person with good vascular function could conceivably be misclassified as having poor vascular function, or vice versa, simply because small variations in the speed of movement.

### Effect of range of motion on PLM‐induced hyperemia

The PLM test of vascular function has primarily been performed moving the leg through a 90° ROM (Gifford and Richardson 2017); however, little justification for using this specific ROM has been published. As illustrated in Figure 4, ROM has a very potent effect on PLM‐induced hyperemia. In contrast with the effect of speed, ROM generally exhibits a curvilinear relationship with the indices of PLM‐induced hyperemia that peaks or plateaus at a ROM of approximately 90°. Indeed, increasing ROM from 30° to 90° resulted in an increase in ΔPeak flow, as well increased AUC_60sec_ (Fig. 4C) and AUC_60cycles_ (Fig. 4D), while increasing ROM beyond the standard 90° actually resulted in a plateauing or decrease in the multiple indices of PLM‐induced hyperemia. As this was also observed when considered the hyperemic response in terms of AUC_60cycles,_ it seems that there is something intrinsic to the ROM that alters the hyperemic response per cycle of movement.

The effect of ROM on antegrade and retrograde flow, which is illustrated in Figure 5A–C, may explain part of the effect of ROM on PLM‐induced hyperemia. Greater flexion of the knee joint (i.e., greater ROM) was associated with increased antegrade ΔPeak and AUC_60cycles_ which reached an apex at 90°. Interestingly, retrograde hyperemia, which exhibited gradual increases in ΔPeak and AUC_60cycles_ between 30° and 60°, exhibited much greater increases in retrograde ΔPeak and AUC_60cycles_ between 60° and 120°. It appears that the increase in retrograde flow when going from 90° to 120° outpaced the change in antegrade flow, preventing any increase in total flow (Fig. 4B–D). That retrograde flow was increased during greater degrees of knee flexion is consistent with a report from McDaniel et al. (2012), who attributed decreased resting leg blood flow in a flexed position to exaggerated retrograde flow in the face of unchanged antegrade flow.

While it is not possible to determine the cause of the increased retrograde flow at greater ROM from the design of this study, evidence from animal‐based studies may shed light on this subject. Using an in vivo preparation with rats, Poole et al. (1997) observed that stretching a muscle to longer lengths resulted in the stretch of the capillaries in the muscle, which, in turn, decreased capillary diameter and resting bulk blood flow by 25–40%. As Kruse et al. (2018) recently reported that muscle fascicle length is positively related to ROM during PLM, it seems possible that the stretch placed on the muscle in the 120° condition resulted in a stretching of the capillary network in the muscle that decreased the diameter of the capillaries, thereby increasing the resistance to flow within the muscle leading to greater retrograde flow and decreased total flow to the muscle.

As illustrated in Figure 6A–D, the relationship between ROM and MAP and heart rate during PLM was determined in a subset of participants (*n* = 9). Importantly, ROM was unrelated to the increase in MAP observed during PLM, indicating that the increased hyperemic response when going from 30° to 120° (Fig. 4A) is not due to a ROM‐induced increase in perfusion pressure. Indeed, as illustrated in Figure 6E–G, the relationship between ROM and the hyperemic response to PLM persists when considering the hyperemic response in terms of vascular conductance, providing additional evidence that the effect of ROM on PLM‐induced hyperemia is specific to the local vasculature.

### Recommendations for the PLM test of vascular function

The PLM test of vascular function is emerging as a useful means of determining the health of the peripheral vasculature (Gifford and Richardson 2017). In this context the impact of variations in movement speed and ROM are quite meaningful. If movement speed or ROM were not tightly controlled during a PLM test this could add a substantial amount of variation, which could make the determination of vascular function difficult. As mentioned, the ~20% change in AUC_60sec_ elicited by a 30°/sec change in movement speed (Figs. 1C, 2C) or the ~35% change in AUC_60sec_ elicited by a 30° change in the ROM could feasibly lead to a person with poor vascular function being classified as normal vascular function and could easily obscure any group differences that exist between populations (e.g., young vs. old; active vs. sedentary) (Gifford and Richardson 2017).

Considering the impact of movement speed and ROM on PLM‐induced hyperemia, it is clear that these factors must be standardized and well‐controlled. Up to this point, most studies have traditionally performed PLM through a 90° ROM at a rate of 180°/sec (Mortensen et al. 2012; Trinity et al. 2012; Gifford and Richardson 2017), but it was previously unknown if this speed and ROM provided the peak response from which to discern the function of the vasculature. In terms of ROM, Figure 4 illustrates that the 90° ROM that has historically been utilized actually provides the greatest PLM‐induced hyperemia of the ROM tested. With the data indicating that deviating the ROM much above or below a 90° ROM would result in an attenuation of PLM‐induced hyperemia, it is clear that the traditionally used 90° ROM is optimal for inducing peak, or nearly peak, hyperemia with PLM.

The data regarding which movement speed provides the optimal response are a little less clear. Figure 1 illustrates that the various indices of hyperemia generally follow a linear relationship with movement speed, increasing with each increase in speed. However, moving the participants’ legs through a 90° ROM at rates greater than 180°/sec (e.g. 240°/sec) was physically difficult to the point that the 240°/sec movement speed seems quite impractical for test administration. In fact, several members of the research team were unable to move the leg through the proper ROM at 240°/sec for more than a few seconds. Moreover, maintaining proper sonography technique during such a rapid leg movement was substantially more difficult than doing so at the traditional 180°/sec. Thus, the traditional 180°/sec movement speed appears to provide the best hyperemic response under practical testing conditions.

While there was initially little published evidence for specifically utilizing a 90° ROM and a 180°/sec movement speed for PLM, the data from this study indicate that this protocol actually appears to provide a near‐peak hyperemic response and optimal testing conditions. With that said, since the near‐peak hyperemia elicited by PLM through a 90° ROM at a rate of 180°/sec has been shown to be related to vascular endothelial function (Mortensen et al. 2012), NO bioavailability (Mortensen et al. 2012; Trinity et al. 2012; Groot et al. 2015; Broxterman et al. 2017) and vascular health (Rossman et al. 2016), we recommend that future studies continue using this traditional protocol (Gifford and Richardson 2017) (PLM through 90° ROM at a rate of 180°/sec) when using PLM to assess vascular function.

### Limitations

The current study was limited to a young, healthy population. It is unknown if different populations may respond to speed and ROM differently. Such a possibility would have very interesting implications for the interpretation of previous studies and warrants further investigation. For example, while the current study demonstrated that the peak hyperemic response in young, healthy individuals occurs with a ROM around the traditional 90°, the traditional 90° ROM may possibly elicit submaximal responses in the elderly. If such is the case, previous data (McDaniel et al. 2010; Mortensen et al. 2012) noting marked differences in endothelial function assessed by PLM‐induced hyperemia between young and old (always 90° ROM) may potentially be confounded by employing a suboptimal protocol for the older individuals. This potential scenario highlights the importance of determining the effect of ROM and speed in multiple populations, despite there being current conformity on the protocol.

## Conclusions

The hyperemic response to PLM is strongly influenced by movement speed and ROM. In general, movement speed exhibits a positive, linear or curvilinear relationship with most indices of PLM‐induced hyperemia. In contrast, ROM generally exhibits a curvilinear relationship with most indices of PLM‐induced hyperemia that peaks around a ROM of 90° of knee flexion. Future studies should be careful to ensure a constant ROM and movement speed when performing PLM. The traditional PLM protocol with a 90° ROM at a rate of 180°/sec actually appears be the optimal protocol for eliciting large hyperemic responses, under practical and feasible test conditions. Therefore, it is recommended that future studies using PLM‐induced hyperemia to assess vascular function continue performing PLM through a 90° ROM at a rate of 180°/sec, under the conditions which have already been shown to relate to many indices of vascular health (Mortensen et al. 2012; Trinity et al. 2012; Groot et al. 2015; Gifford and Richardson 2017).

## Conflict of Interest

The authors have no conflicts of interest to report.
